# How older patients prioritise their multiple health problems: a qualitative study

**DOI:** 10.1186/s12877-019-1373-y

**Published:** 2019-12-21

**Authors:** Ulrike Junius-Walker, Tanja Schleef, Ulrike Vogelsang, Marie-Luise Dierks

**Affiliations:** 1 Institute of General Practice, Hannover Medical School, Carl-Neuberg-Str. 1, 30625 Hannover, Germany; 2 Institute for Epidemiology, Social Medicine and Health Systems Research, Hannover Medical School, Carl-Neuberg-Str. 1, 30625 Hannover, Germany

**Keywords:** Older adults, Health priority setting, Multimorbidity, Qualitative research

## Abstract

**Background:**

Patients with multimorbidity often receive diverse treatments; they are subjected to polypharmacy and to a high treatment burden. Hence it is advocated that doctors set individual health and treatment priorities with their patients. In order to apply such a concept, doctors will need a good understanding of what causes patients to prioritise some of their problems over others. This qualitative study explores what underlying reasons patients have when they appraise their health problems as more or less important.

**Methods:**

We undertook semi-structured interviews with a purposive sample of 34 patients (aged 70 years and over) in German general practices. Initially, patients received a comprehensive geriatric assessment, on the basis of which they rated the importance of their uncovered health problems. Subsequently, they were interviewed as to why they considered some of their problems important and others not. Transcripts were analysed using qualitative content analysis.

**Results:**

Patients considered their health problems important, if they were severe, constant, uncontrolled, risky or if they restricted daily activities, autonomy and social inclusion. Important problems often correlated with negative feelings. Patients considered problems unimportant, if they were related to a bearable degree of suffering, less restrictions in activities, or psychological adjustment to diseases. Altogether different reasons occurred on the subject of preventive health issues.

**Conclusions:**

Patients assess health problems as important if they interfere with what they want from life (life values and goals). Psychological adjustment, by contrast, facilitates a downgrading of the importance. Asking patients with multimorbidity, which health problems are important, may guide physicians to treatment priorities and health problems in need of empowerment.

## Background

Multimorbidity in older patients frequently requires general practitioners to prioritise treatments [1]. Yet due to time constraints, individual consultations are often directed towards managing a single chronic condition. Other conditions are dealt with in later consultations leading to treatments for concomitant health conditions which are poorly synchronized [2].This has implications for patients, in terms of overall heavy treatment burden and medication safety [3, 4].

Care planning in the face of multiple morbidities requires decisions on what and what not to treat. An “expert panel on the care of older adults with multimorbidity” recommends eliciting individual health goals as a guiding principle for these challenging decisions [5]. “Health goals” are defined here as broad health and life outcomes that people hope to gain or maintain (e.g. everyday function) [6]. The term “health priorities”, in contrast, refers to the result of individual treatment decisions on the basis of conflicting treatment choices that occur with multimorbidity. Health priorities, ideally, emerge as a result of person-centered care planning processes, in which patients reflect and reveal what matters to them. However, in practice all too often the medical perspectives of physicians guide the decision on what to treat [7]; and it has become evident that physicians tend to focus on clinical aspects whereas patients primarily consider everyday life effects [8, 9]. The different perspectives result in substantial disagreements on health and treatment priorities [10, 11], which makes it even more important to include patients’ views when setting priorities in a care planning process.

Older patients’ views on what really matters to them in terms of health are largely unexplored. A qualitative study examines health priorities of different patient populations (osteoarthritis, multimorbidity) and determines that conditions with underlying functional impairments and the risk of future dependency matter most [4]. In another qualitative analysis patients prioritise worrisome illnesses, complications and potentially disabling diseases [12]. A discrete choice experiment reveals that priorities vary according to personal circumstances and life values [13]. The few studies on life goals and values in this age group convey that it is important to feel well (including managing symptoms and body functions), to be connected, to make use of one’s capabilities, to be independent from the support of others, and to construe personal life as sense-making [8, 14, 15].

The aim of this paper is to explore what underlying reasons patients have when they assess the importance of their health problems. The findings shall facilitate an enhanced understanding on principles of patients’ health priority decisions in the face of multiple morbidities competing for treatment. Unlike previous studies, patients evaluate their personal priorities based on findings from a comprehensive geriatric assessment assuring that a wide range of their problems are assessed whether deemed important or not.

## Methods

Eight general practices in the region of Hannover approached altogether 48 patients and explained the study. The desk nurses addressed consecutive patients visiting the practice on a predefined date. The purposive sampling took age (70–79 years, 80 and over) and an equal gender ratio into account. Exclusion criteria were: living in a care home, receiving nursing care support (stage 2, 3), severe dementia, and problems with communication. Participating patients gave written consent. The study was approved by the ethics committee, Hannover Medical School (No. 5069).

The study patients first underwent a comprehensive geriatric assessment (STEP) in the practices. STEP assesses 44 health conditions and every day life problems in the domains of function, social health, medical problems, mood, life-style, immunization, medication, cognition. Additional health conditions are also determined. Immediately afterwards, the participants received a computer-generated list of their findings. They then rated their disclosed health problems according to importance: not important, slightly important, quite a bit important, or very important.

After a few days the patients were visited in their homes to engage in a semi-structured interview. They were asked to expand on the disclosed health problems and the importance for their health and daily life [16]. Interviews lasted on average 29 min (range 10–50).

The method of qualitative content analysis by Schreier was used for analysis [17]. First, we selected text material that dealt with the importance of a health problem. A coding frame was developed with the themes “important”, “unimportant” and “both, important and unimportant” as well as “type of health problem” as main categories. Subcategories were generated as data driven codes and arranged under the main categories. Subcategories could be only coded once under one main category to assure reflective demarcation between subthemes. Each subcategory received an explanation, indicators and a coding example to guide the coding procedure. In this way, two investigators (UJW and UV) developed the coding frame using two interviews. TS and UV then independently piloted four further interviews with an overall 87% agreement of coding. Differences in codings were discussed and the coding frame altered. Using the final coding frame, UV then coded all 34 interviews. Mindmaps were created for each main category to explore cross-thematic findings in the subcategories.

## Results

35 patients (participation rate 73%) were recruited in eight general practices. One patient dropped out after the assessment. The remaining 34 participants were on average 77.8 years old. The assessment disclosed on average 17.2 health problems. Table 1 informs about the patients’ characteristics and the number of their uncovered and rated health problems. In the following interviews, patients then explained for altogether 392 health problems why they assess them as important, unimportant or both.

**Table 1:** Participants’ characteristics, health problems uncovered in STEP and problem ratings

	all (*N* = 34)		female (*N *= 17)		male (*N* = 17)	
education level^x^, N(%)						
- low - medium - high	7 21 6	(21) (62) (18)	6 11 0	(35) (65) (0)	1 10 6	(6) (59) (35)
age (yrs) mean (stdev)	77.8	(+ − 5.3)	79.1	(+ − 5.5)	76.5	(+ − 4.9)
HP uncovered*, mean (stdev)	17.2	(+ − 8.5)	21.7	(+ − 8.9)	12.7	(+ − 5.3)
HP*, grp ^ϒ^ < 80 yrs., mean (stdev)	16.4	(+ − 8.5)	19.9	(+ − 10.2)	13.0	(+ − 4.8)
HP *, grp ^ϒ^ ≥ 80 yrs., mean (stdev)	18.1	(+ − 8.8)	23.8	(+ − 7.4)	12.4	(+ − 6.0)
HP rated**, mean (stdev)	11.2	(+ − 5.0)	13.8	(+ − 5.3)	8.7	(+ − 3.1)
HP **, grp ^ϒ^ < 80 yrs., mean (stdev)	10.6	(+ − 4.3)	12.7	(+ − 4.1)	8.4	(+ − 3.4)
HP **, grp ^ϒ^ ≥ 80 yrs., mean (stdev)	11.9	(+ − 5.8)	15.0	(+ − 6.4)	8.9	(+ − 2.9)
HP** rated important mean (stdev)	6.9	(+ − 4.4)	8.9	(+ − 5.0)	4.8	(+ − 2.2)
HP** rated unimportant mean (stdev)	3.3	(+ − 1.9)	3.7	(+ − 2.2)	2.9	(+ − 1.4)
HP** import & unimport. mean (stdev)	1.1	(+ − 1.0)	1.2	(+ − 1.0)	0.9	(+ − 1.0)

^X^education: low: primary school without vocational training,

middle: vocational training completed,

high: academic qualification

^ϒ^age group (grp)

*number of health problems (HP) uncovered in the STEP assessment

**number of health problems (HP) discussed and rated in the interview after the assessment

### Reasons, why health problems are deemed important

**Fig. 1:**
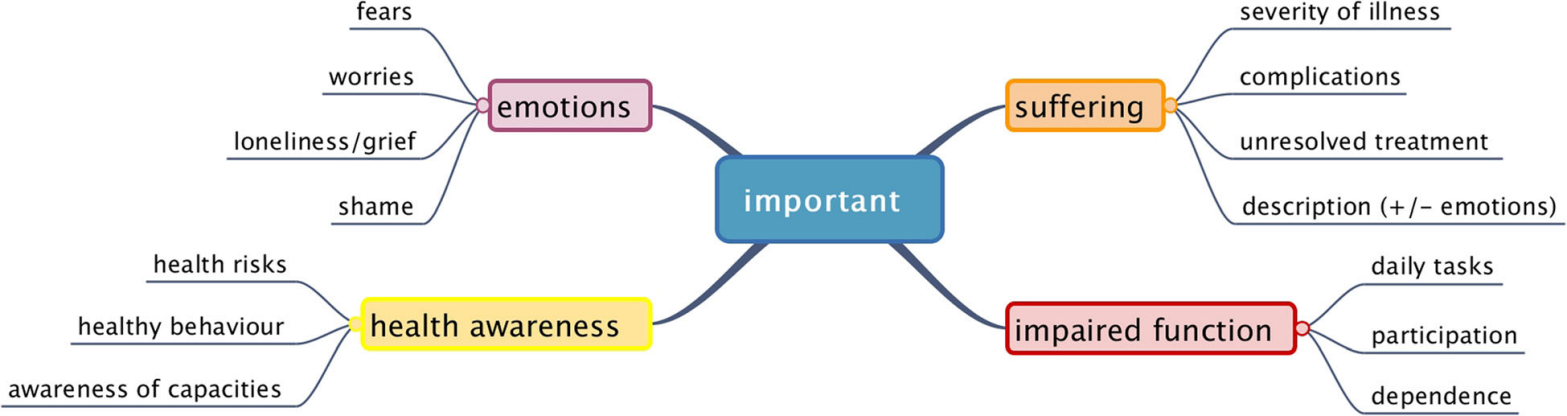
Reasons for important health problems

Altogether, patients gave explanations for 245 important health problems. Four main themes emerged, in which explanations were assigned to further subcategories (see Fig. 1).

Suffering was a major reason for an important health problem. Suffering had different meanings for patients. First of all, patients suffered, if an illness was deemed severe. A severe illness was experienced as intense or deteriorating, chronically present, or as having negative effects on daily activities.

Suffering also occurred due to medical complications. Falls were a frequent example. *P29/4 “I have fallen so often because of my eyes, and I have broken a tooth because of this fall.”*

In other cases, patients linked their suffering with ineffective treatments. This meant they had experienced little improvements or side effects; and they felt resigned. *P6/17 “Yes, this is also very important, of course. I need a hearing aid for both ears; but when I eat something, I don’t hear anyway, I hear me eating. …And when there is something on television or somebody talks to me, I cannot hear it anyway although I wear hearing aids.”*

Different types of health problems were associated with suffering such as pain, difficulty sleeping, dysaesthesia, incontinence, chest pain, falls and hearing impairment.

Underlying functional impairments were also rated important. In the first instance, patients felt compromised managing their daily tasks. They explained how difficult it was to provide themselves with food, to keep the flat clean and to attend to personal care. Patients also talked about how mobility problems made their lives difficult, especially climbing stairs. Patients sometimes lamented the lack of energy that forced them to take breaks. *P1/5 “I have to take a break on every flight of stairs. When I am up here, I think I am broken”.* Sometimes vital self-management became burdensome, like having to stay close to a toilet in the case of incontinence. Others could not keep up their daily routines anymore. In some cases, unmet needs became apparent. *P32/1 “This helplessness, which I have due to the vertigo. This limits me in many things, which I cannot do anymore… where I need help”.*

Functional impairments led to a restricted participation with the outside world. Patients bemoaned the loss of going for a walk, pursuing hobbies and meeting friends. *P8/5 “Going for a long walk - this I cannot do. I only walk to the [nearby] cemetery. …Then I take my trolley and I can rest before I go home again.”*

Other consequences were social exclusion or dependence. *P6/9 “[It is important for me] that I could live without help. I always need, always I need help; and for me it would be very, very important, if I was able to live without help.”*

Functional impairments occurred due to very different underlying medical problems, e.g. pain, incontinence and sensory impairments. They restricted self-care, activities of daily living, autonomy and social participation and were therefore considered important.

Thirdly, negative feelings were expressions of important health problems. Fear was a predominant theme. Patients had either experienced life threatening illnesses (e.g. myocardial infarct, cancer, adverse drug reaction) and feared recurrence or they simply feared a deterioration of their condition (e.g. cardiovascular condition, fear of falling, looming operation). *P28/1 “My leg can get worse, because of the lack of blood flow…and of course I ponder over the future, how it will be for me… how it develops...”*

Some patients were preoccupied with worries directed towards the health of the partner, towards loosing independence, towards harmful polypharmacy or the financial situation. Other patients felt ashamed because of their incontinence. Frustration arose in connection with hearing impairments. Patients expressed loneliness and grief, when having lost functional independence or when suffering from the death of a close person.

*P46/2 “One has many lonely hours... First the children gone, then the work gone, then the husband gone, well, these are decisive steps in life.”*

General anxiety and depression were also sometimes present but not further explained.

Health awareness was the fourth main topic linked to the importance of health problems. Patients were aware that it is important to know about cardiovascular risks, about the protective properties of vaccinations and to take medications as prescribed.

*P7/8 “I always think, if this [cholesterol level] is too high, it is never ever good; well, one should actually take an interest in it.”*

### Reasons, why health problems are deemed unimportant

**Fig. 2:**
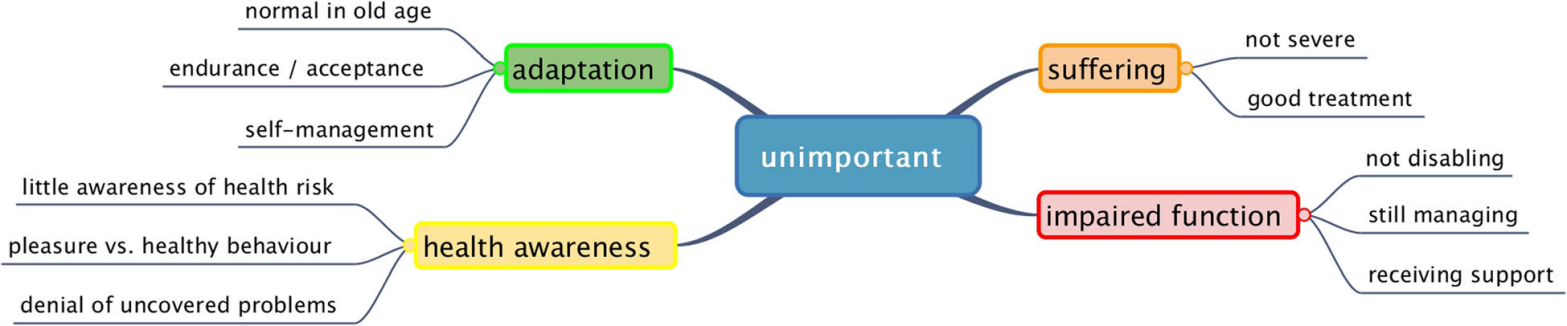
Reasons for unimportant health problems

The participants also gave reasons for 112 out of 392 health problems that were deemed unimportant. They were often the opposite reasons given for important health problems. However, adaptation emerged as a new theme (see Fig. 2).

Little or no suffering was a reason why patients evaluated their problems as rather unimportant. These problems were either well treated or not severe. Non-severe problems, in the eyes of the patients, were those that of short duration, intermittently present, not (always) intense, improving, or were not linked to medical complications.

Good professional care relieved patients from suffering and worrying. In such cases they explained that the doctor monitors or treats their problems well, that they have adopted the doctor’s opinion on the irrelevance of the problem or that they rely on the doctor and trust her/his expertise. *P2/2 (Multimedication) “I prefer to lay this into her [the doctor’s] hands and trust her….”*

Little negative impact on the daily lives made patients rate their problem as unimportant. “I can still manage” was the catchphrase. Some patients explained that their impairment was not (yet) disabling; others talked about having got used to the inconvenience of their disability and still managed that way. *P27/5 “Well, surely; I am not so flexible any more, but I still manage to climb in a bath tub”.*

If patients received reliable support for tasks they could not perform, they also rated them as rather unimportant. P35/3 *“I have my dear Monica… She already came here when my husband was still alive, and she often took this [going shopping] off my hands. Sometimes I go with her, sometimes not, it depends.”*

Little health awareness led patients to assess preventive health problems as unimportant. Subthemes covered the irrelevance of vaccinations, the negligence of healthy behaviour and denial of incapabilities.

Vaccinations were rated as unimportant because the risk of acquiring infections was assessed as small. Patients thought that vaccinations were meant to protect childhood diseases or would last for a lifetime. Some patients said that in their long life they never caught such an infection and found it unlikely to acquiring it in the future. One patient feared that vaccinations are poisonous. Interestingly, many explanations were phrased as questions, which emphasize the lack of certainty in this field. *P4/10 “Diphtheria – can you still get it in old age? Isn’t this a childhood disease? I think, that this is not necessary. If you have so many drugs and foreign matter pumped into your body- so many vaccinations – is this good?”*

Some patients admitted to an unhealthy diet. The pleasure of eating unhealthy foods was more important than the preventive aspects of less tasty but healthy foods. One patient felt reassured because his diet has not harmed him throughout his long life. *P13/3* “*Well, I do not much take care about…I always eat fat; I also eat many vegetables. I eat what I want.”*

Some health problems were newly uncovered in the geriatric assessment and deemed unimportant at first, such as a low cognitive performance, hypothyroidism and in one case diabetes. *I:* “*And the clock drawing [cognitive test]. Is this important to you?* “*P30/15 „No, not really. I only thought afterwards: Oh, what have you messed up? The ‘12`, the ´6`. It belongs here, doesn’t it?”*

Adaptation emerged as another theme explaining the unimportance of health problems. Firstly, patients interpreted their declining body functions as inevitable ageing processes.

*P3/5* “*If one has difficulties getting up… but this is totally normal, or is it not? I cannot jump like a young deer”.*

Secondly, patients came to terms with their problems. There was a continuum from “enduring” the problem to accepting it. *P14/6 (Dizziness) “Yes. For me this is not [important] – I have to live with it now.”*

The third aspect of adaptation comprised active self-management strategies. Patients found ways how to successfully deal with their problems. Others felt assured that the doctor monitored their problem(s) or had given advice on what to do in case of deterioration. *P37/2 (Back pain). I: “And do you manage”? P:” Yes, hot water bottle, ABC-[heat]-plaster and massage from the wife.”*

### Reasons, why health problems have important and unimportant aspects

Half of the patients had at least one health condition (altogether *n* = 35), which they found difficult to classify. Four patterns emerged. Firstly, health problems were thought important in general but not personally. These problems were mainly cardiovascular conditions or vaccinations. Secondly, patients explained that their problem had been important in the past, but symptoms had improved. Again, cardiovascular problems prevailed. A third pattern related to taboo subjects, such as hearing difficulty, incontinence and cognitive deficits. Patients admitted in the interview that on second thought these problems were important after all. Lastly, some patients evaluated their problems as rather important but felt that they could cope. *P7/2 “Well one does not always sleep evenly….One is unable to liberate oneself from this [ruminating thoughts]. But then I have an afternoon nap”.*

Cross-thematic comparison revealed that constant effects of medical conditions and negative feelings were often found across important categories (negative feelings, suffering, and impaired functions). Being able to manage and psychological adjustments were found across unimportant categories.

## Discussion

34 older patients evaluated the importance of their multiple health problems disclosed by a geriatric assessment. When deciding upon the importance of specific health issues, the patients considered the broader impact on their lives rather than disease specific aspects.

### Reasons for assessing health problems as important

The patients find their chronic conditions important, if they are associated with substantial physical, emotional or functional strains. A high symptom burden or prospect of medical complications contribute to physical strain. Impaired functions impact on daily lives, in particular on mobility, independence, self-sufficiency and social relations. Negative feelings accentuate the importance of a problem.

A qualitative study examining priority decisions on competing outcomes of patients with polypharmacy reports similar findings. It is suggested that symptom burden, physical function and survival are the main categories for older patients that determine the decision making in the priority setting process [18]. The WHO similarly advocates five broad underlying values or goals that guide people in their decisions: to be mobile, having relationships, meeting basic needs, learning/growing and making decisions, and contributing [19]. In addition, our study findings point to the relevance of emotional experiences that accompany chronic conditions. Negative feelings seem to consistently contribute towards an appraisal of importance; they may reveal more or less hidden clues of help seeking behaviour [20].

### Reasons for assessing health problems as unimportant

Unimportant problems are often either not as severe in nature or appear related to psychological adjustments. Adjustments are made externally, e.g. receiving support from professionals or relatives, and internally. One type of internal adjustment is *cognitive.* It includes sense making and recalibration. That is evident from the way patients talk about their individual genetic risks and pathogenesis or explain their physical deterioration as a natural phenomenon of ageing. *Cognitive-emotional adjustments* have also been revealed*.* It seems that some patients approach their negative health related feelings and modulate their emotions. This is expressed in a continuum from enduring to accepting the strains caused by illnesses. Avoidance has also been observed. Whereas avoidance is generally perceived as a mal-adaption, it can help in early stages of change or in non-severe diseases [21]. A third type of adjustment is *active and problem-focused.* Some of our patients have talked about managing their health related issues by having altered their lifestyle, having found ways of self-help or support, and by monitoring/taking control of the disease. Due to these adjustments patients downgrade the importance of their problems, a mental approach that has been described previously [22–24]. A recent qualitative study on personal strengths has identified strategies that patients apply to handle their chronic diseases. Whereas external strengths entail receiving support, internal strengths include being positive and kind, reconciling oneself with the situation, being knowledgeable and in control, and having courage [25]. Cognitive emotional resources and adjustments have also been identified in another interview study with older patients [26].

Finally, health awareness has emerged as a category that contrasts the previous reasons for importance and unimportance. In this case, unimportant health problems are associated with little health awareness (especially vaccinations) and require physicians’ attention. Reasons are often lack of information and risk perceptions [27].

## Strengths and limitations

This study presents reasons that patients with multimorbidity have when deciding upon health priorities. One strength of our study design is that health problems are determined by a comprehensive geriatric assessment and represent a large spectrum of diseases, functional impairments, psychological, mental and social issues. Furthermore patients considered their own problems – thereby avoiding priority setting on a general list of health problems that are not personally experienced.

The limitations of the study must also be considered. The importance of individual health problems was discussed with patients of different educational background. It remains unclear in what way the educational level influenced rating results and ease of deciding on an importance level. Further studies are needed to determine the robustness of the ratings. Additionally, patients’ perceptions on their health problems only represent a “snapshot” in time. There is evidence that preferences change over time and that re-prioritisation takes place with changing illnesses and circumstances [28, 29]. Due to the abundance of health problems, patients did not explain all importance ratings, and explanations sometimes were quite short and prohibited an in depth analysis. Results are restricted to patients living in the community with no need of nursing care or with a designation into the lowest nursing care level.

## Assessing the importance of health problems: implications for practice

As yet, only few recommendations exist on how to practically conduct priority setting decisions with older patients [30]. The American Geriatrics Society [5] suggests to *indirectly* consider patient preferences by “asking patients to prioritize a set of universal health outcomes…(e.g., living as long as possible, maintaining function…)” so that treatment options can be prioritized according to these preferred universal health outcomes. The same indirect strategy is recommended in an earlier publication [31]. Naik et colleagues have published a set of questions that elicit five distinct health related life values suitable for such purpose [14]. Also, European organisations recommend individualized care plans on the basis of obtaining universal patient preferences [32, 33]. In the Netherlands a strategy was tested, in which older patients weighed up the relevance of four universal health outcomes against each other (longevity, independence, reduction of pain and symptoms) [34]. Our findings indicate that another approach may be possible, that is to *directly* elicit from patients which of their health problems are important. Since patients seem to automatically assess the problems according to these universal health outcomes or life goals, they fall back on the same underlying priority setting principle.

## Conclusions

Considering the growing number of older people, efforts are directed towards responding to challenges associated with chronic and multiple health care needs [35]. One shift in orientation is to reformulate treatment goals towards supporting resilience and self-management of incurable diseases [36]. The other shift involves a priority setting by placing important health conditions over less important ones. What is important is all too often impossible for doctors to determine on medical grounds alone. Asking patients with multiple diseases, which of their health problems are important to them, may guide physicians to both – patient centred priority issues and specific areas in need of empowerment.

## Data Availability

The datasets used and/or analysed during the current study are available from the corresponding author on reasonable request.

## Abbreviations

HP: health problems
STEP: standardised assessment for the elderly in primary care
